# A statistical method to incorporate biological knowledge for generating testable novel gene regulatory interactions from microarray experiments

**DOI:** 10.1186/1471-2105-8-317

**Published:** 2007-08-29

**Authors:** Peter Larsen, Eyad Almasri, Guanrao Chen, Yang Dai

**Affiliations:** 1 Core Genomics Laboratory at University of Illinois at Chicago, 845 West Taylor Street Chicago, IL 60607, USA; 2 Department of Bioengineering (MC063), University of Illinois at Chicago, 851 South Morgan Street, Chicago, IL 60607, USA; 3 Department of Computer Science, University of Illinois at Chicago, 851 South Morgan Street, Chicago, IL 60607, USA

## Abstract

**Background:**

The incorporation of prior biological knowledge in the analysis of microarray data has become important in the reconstruction of transcription regulatory networks in a cell. Most of the current research has been focused on the integration of multiple sets of microarray data as well as curated databases for a genome scale reconstruction. However, individual researchers are more interested in the extraction of most useful information from the data of their hypothesis-driven microarray experiments. How to compile the prior biological knowledge from literature to facilitate new hypothesis generation from a microarray experiment is the focus of this work. We propose a novel method based on the statistical analysis of reported gene interactions in PubMed literature.

**Results:**

Using Gene Ontology (GO) Molecular Function annotation for reported gene regulatory interactions in PubMed literature, a statistical analysis method was proposed for the derivation of a likelihood of interaction (LOI) score for a pair of genes. The LOI-score and the Pearson correlation coefficient of gene profiles were utilized to check if a pair of query genes would be in the above specified interaction. The method was validated in the analysis of two gene sets formed from the yeast Saccharomyces cerevisiae cell cycle microarray data. It was found that high percentage of identified interactions shares GO Biological Process annotations (39.5% for a 102 interaction enriched gene set and 23.0% for a larger 999 cyclically expressed gene set).

**Conclusion:**

This method can uncover novel biologically relevant gene interactions. With stringent confidence levels, small interaction networks can be identified for further establishment of a hypothesis testable by biological experiment. This procedure is computationally inexpensive and can be used as a preprocessing procedure for screening potential biologically relevant gene pairs subject to the analysis with sophisticated statistical methods.

## Background

Microarrays are routinely used to simultaneously assess the relative expression levels of many thousands of gene transcripts in biological samples. Increasingly sophisticated techniques have been developed to detect those transcripts among thousands whose expression levels have significantly changed in response to the selected experimental conditions. Yet these lists of differentially expressed genes, however highly refined, are of limited use in themselves. A deeper understanding of the underlying biological mechanisms depends on the identification of how these genes are interacting with the cell. Through the development of gene interaction networks, microarray data evolve from being descriptive to being predictive.

The use of various statistical methods for identifying significant transcriptional regulatory interactions and for uncovering the corresponding regulatory network structures is a major step toward that direction [1-13]. However, microarray data themselves do not provide enough information for the definitive identification of regulatory interactions within the cell. The inclusion of other biological information is essential. Recently, several groups have proposed methods of constructing regulatory networks based on microarray gene expressions and other large sets of high throughput data [14-16]. Particularly, the methods in [15,16] can perform analysis of a highly diverse collection of genome-wide data sets, which include gene expression, protein interactions, growth phenotype data, and transcription factor binding, to reveal the modular organization of the yeast system.

The identification of regulatory networks at genome scale is important. However, it does not address the issue of analyzing a single set of microarray data obtained from a specific hypothesis-driven experiment. It is possible that the network structure derived based on the above method may be not all observed in a single microarray experiment that was conducted under specific experimental conditions. Therefore, it remains an important task for individual investigators to identify the interactions that are most consistent with the data. There are several databases to which a set of genes can be submitted for querying of possible pathways. However, the discovery of new pathways is often not possible by using these databases. This situation has become a major obstacle for deriving new testable hypotheses from a single set of microarray data conducted by an individual research group.

On the other hand, the large body of information hidden in the published literature has not been effectively integrated in the microarray data analysis. Several groups [17-19] have attempted to utilize prior biological knowledge to substantially reduce false positives in the outcomes from the data-driven approaches. Herrgard *et al *. [18] have evaluated networks reconciled from gene expression data, known genome-scale regulatory network structures generated from annotated genome information, well-curated databases, and primary research literature. Jenssen *et al. *[20] proposed a gene-to-gene co-citation network for 13,712 named human genes by an automated analysis of titles and abstract in over 10 million MEDLINE records. PathwayAssist 3.0 analysis software by Ariadne Genomics [21] also provides networks for a set of query genes based on a natural language search algorithm of all available PubMed published abstracts. The limitation of these methods is their inability of discovering novel interactions, since the reported interactions in literature are often used to overlap with the interactions identified from the analysis of microarray data.

Ideally, a method for determining gene interaction networks would have the ability to (1) use the large amount of prior biological knowledge available to researchers and (2) identify novel gene interactions in a set of microarray expression data for a specific study. The method proposed here attempts to address these two requirements. Our method is designed to identify small number of testable hypotheses from a specific microarray data set and collected biological observations.

In order to evaluate our method, we define the type of gene interaction considered in this paper as follows. As microarray experiments measure changes in gene expression, the gene interaction used here will be restricted to what might be reasonably expected to be observed in a microarray experiment: a change in the expression of a regulator gene modulates the expression of a target gene. These pairwise interactions form the gene interaction network of a particular system under a particular condition.

## Results

### Datasets

The datasets used in this study are subsets of cell-cycle dependent genes in the budding yeast Saccharomyces cerevisiae microarray experiments [22]. These microarray experiments were designed to create a comprehensive list of yeast genes whose transcription levels were expressed periodically within the cell cycle. Microarrays were prepared for this study by printing PCR-generated probes. In order to study cell cycle related gene expression, the cell cycles of yeast cultures needed to be synchronized. A sample of growth media would contain cells all in the same stage of the cell cycle. The study used for synchronization of the cell cycle is alpha-factor based. Alpha-factor, a signaling pheromone, causes cells to undergo cell cycle arrest. Pelleting the cells and re-suspending them in fresh media permits normal cell cycle to continue. For the alpha-factor experiment, the gene expressions of cell cycle synchronized yeast cultures were collected over 18 time points taken in 7-minute intervals. This time series covers more than two complete cycles of cell division.

Two subsets of the data were selected for our study. The first is a subset of 102 genes that includes 10 known transcription regulators and their possible regulatory targets [23]. This set is highly enriched for known interacting genes involved in the Saccharomyces cell cycle.

The second subset is comprised of 999 of the most cyclically regulated genes in the microarray experiments. In the analysis of a hypothesis-driven experiment, it is neither necessary nor logical to investigate every gene in the genome of an organism. It can be expected that the majority of genes in the genome are irrelevant to the biological phenomenon under investigation. It might be reasonable, for example, to restrict analysis to those genes observed to undergo statistically significant changes or genes that are detected as being significantly expressed. As the data were collected in an experiment to observe genes that are instrumental to directing cell cycle activities, only those genes identified as being cyclically expressed will be used in our analysis. Though there is a number of methods that have been used in the past for finding cyclic expression patterns [24-27], the method used here most resembles that of Filkov *et al *. [28]. In this, stretches of gene expression data are compared against themselves to look for recurring patterns of expression. This was carried out through the computation of the cyclic correlation coefficient (CCC) of a gene. See Methods section for the definition. The 999 genes with the highest CCC were selected for analysis. The expressions of the genes were identified to fit well with their expected phase in the mitotic cycle. The annotations of these genes were identified to be enriched for annotations specific to cell cycle.

### Identify gene interaction pairs from the likelihood of interaction scores and the modified Pearson correlation coefficients

One of the goals of this study is to identify gene pairs that are likely to interact on the basis of prior biological knowledge. The likelihood of interaction (LOI) score is the result of efforts to achieve this goal. Using prior information in the PubMed database of scientific publications, information about previously observed gene interactions was collected and used to generate an LOI-score for a gene pair. This score was used to determine if a gene pair is likely a potential interaction pair or not (for details see Methods). If the gene pair closely resembles gene interaction pairs frequently observed in the literature, it is considered likely and should have a high LOI-score; if the gene pair has little similarity to previously observed gene interactions, their interaction is considered unlikely and should have a negative LOI-score. LOI-scores for all possible pairs of genes from a set of query genes obtained from an experiment will be assigned and arranged in an LOI-score matrix. An excel template of the program for calculating LOI-scores was made available [29].

On the other hand, a modified Pearson correlation coefficient (PCC) was used to identify the possible gene interaction pairs from the microarray data. A regulation interaction defined in this genetic network is a gene whose change in expression levels has an effect on the subsequent expression of another gene. The 1-time point shift of the putative target gene relative to the regulator gene used here identifies this type of causal relationships in a simplified way.

Two sets of possible gene interactions are considered significant after the multiple hypothesis testing: one obtained from LOI-scores and the other one obtained from PCCs. The former set is determined by our prior biological knowledge on a given set of genes collected from scientific literature. The latter set includes statistically significant interactions identified from the analysis of the microarray data from a specific hypothesis-driven experiment. The overlap of these two sets forms a network of gene interactions. These interactions in the network were analyzed to determine how many of the previously published interactions were identified, as well as the number of gene interaction pairs in which both the regulator and the target sharing at least one Biological Process (BP) annotation as provided by the Gene Ontology (GO) Slim Mapper [30] in Saccharomyces Genome Database. Good gene interaction networks are expected to be enriched for both previously published gene interactions and interaction pairs that share GO BP annotations.

Results of using different thresholds *q * for false positive rate (fdr) [31] for each set, denoted by LOI.fdr(*q *) and PCC.fdr(*q *) for LOI-scores and PCCs respectively, and their common sets with different thresholds are presented below.

### 102-gene set

The results for the 102-gene cell cycle set are summarized in Table 1. At PCC.fdr(0.05), 1377 regulatory interaction pairs are identified, 30 (2.3%) of which had been previously reported in an automated survey of the scientific literature, and 526 (38.2%) interaction pairs share at least one GO BP annotation between the regulator and the target. At LOI.fdr(0.05), 2,237 regulatory interaction pairs are identified, 142 (6.3%) of which have been previously reported, and 809 (36.2%) of the identified interaction pairs share a GO BP annotation between the regulator and target. Combining LOI.fdr(0.05) and PCC.fdr(0.05), the method generates a network of 289 interaction pairs, 22 (7.6%) of which have been previously reported and 114 (39.4%) of the identified interaction pairs share a GO BP annotation between the regulator and target.

**Table 1:** Results for the 102-gene set

	**LOI.fdr(q)**	**PCC.fdr(q)**	**Number of Edges Identified**	**Published Edges**	**Same GO BP Annotation**	**% Published**	**% Same GO-BP**
A	**GO-simple**	**NoRestriction**	**9147**	**171**	**2418**	**1.9**	**26.6**

B	NoRestriction	PCC(0.1)	1701	37	633	2.2	37.2
	NoRestriction	PCC(0.05)	1377	30	526	2.2	38.2
	NoRestriction	PCC(0.005)	346	12	156	3.5	45.1

C	LOI(.1)	NoRestriction	2312	142	840	6.1	36.3
	LOI(0.05)	NoRestriction	2237	142	809	6.3	36.2
	LOI(0.005)	NoRestriction	1976	136	577	6.9	29.2

D	LOI(.1)	PCC(0.1)	354	26	138	7.3	39.0
	LOI(0.05)	PCC(0.1)	345	26	133	7.5	38.6
	LOI(0.005)	PCC(0.1)	308	26	101	8.4	32.8

E	LOI(.1)	PCC(0.05)	298	22	119	7.4	39.9
	**LOI(0.05)**	**PCC(0.05)**	**289**	**22**	**114**	**7.6**	**39.4**
	LOI(0.005)	PCC(0.05)	257	22	87	8.6	33.9

F	LOI(.1)	PCC(0.005)	69	10	32	14.5	46.4
	LOI(0.05)	PCC(0.005)	68	10	31	14.7	45.6
	**LOI(0.005)**	**PCC(0.005)**	**64**	**10**	**29**	**15.6**	**45.3**

**A: GO-simple**: a pair of genes is considered potentially interacting if one of their GO annotation pairs has been reported.

**B: **set of interactions obtained by applying only PCC thresholds;

**C**: set of interactions obtained by applying only LOI thresholds;

**D**: common set of interactions obtained by applying different LOI thresholds and a fixed PCC.fdr(0.1);

**E**: common set of interactions obtained by applying different LOI thresholds and a fixed PCC.fdr(0.05);

**F**: common set of interactions obtained by applying different LOI thresholds and a fixed PCC.fdr(0.005).

### 999-gene set

Results for the set of 999 cyclically expressed genes are summarized in Table 2. At PCC.fdr(0.05), 5,987 regulatory interaction pairs are identified, 27 (0.45%) of which had been previously reported in an automated survey of the scientific literature, and 813 (13.6%) interaction pairs share at least one GO BP annotation between the regulator and the target. At LOI.fdr(0.05), 95,222 interaction pairs are identified, 224 (0.24%) of which have been previously reported, and 12,963 (13.6%) of the identified interaction pairs share a GO BP annotation between the regulator and target. The set of interaction pairs with an LOI.fdr(*q *) less than 0.005 shows slight improvement over that of LOI.fdr(0.005). Combining LOI.fdr(0.05) and PCC.fdr(0.05) in the 999-gene set, the method generates a network of 757 interaction pairs, 15 (2.0%) of which have been previously reported and 174 (23.0%) of the identified interaction pairs share a GO BP annotation between the regulator and target, a significant increase.

**Table 2:** Results for the 999-gene set

	**LOI.fdr(q)**	**PCC.fdr(q)**	**Number of Edges Identified**	**Published Edges**	**Same GO BP Annotation**	**% Published**	**% Same GO-BP**
A	**GO-simple**	**NoRestriction**	**730688**	**641**	**60691**	**0.09**	**8.3**

B	NoRestriction	PCC(0.1)	18350	62	2280	0.34	12.4
	NoRestriction	PCC(0.05)	5987	27	813	0.45	13.6
	NoRestriction	PCC(0.005)	931	4	144	0.43	15.5

C	LOI(0.1)	NoRestriction	96742	226	13220	0.23	13.7
	LOI(0.05)	NoRestriction	95222	224	12963	0.24	13.6
	LOI(0.005)	NoRestriction	90875	202	12326	0.22	13.6

D	LOI(0.1)	PCC(0.1)	2196	25	468	1.14	21.3
	LOI(0.05)	PCC(0.1)	2145	25	453	1.17	21.1
	LOI(0.005)	PCC(0.1)	2053	23	434	1.12	21.1

E	LOI(0.1)	PCC(0.05)	781	15	177	1.92	22.7
	**LOI(0.05)**	**PCC(0.05)**	**757**	**15**	**174**	**1.98**	**23.0**
	LOI(0.005)	PCC(0.05)	733	15	170	2.05	23.2

F	LOI(0.1)	PCC(0.005)	163	2	48	1.23	29.4
	LOI(0.05)	PCC(0.005)	159	2	48	1.26	30.2
	**LOI(0.005)**	**PCC(0.005)**	**153**	**2**	**48**	**1.31**	**31.4**

**A: GO-simple**: a pair of genes is considered potentially interacting if one of their GO annotation pairs has been reported.

**B: **set of interactions obtained by applying only PCC thresholds;

**C**: set of interactions obtained by applying only LOI thresholds;

**D**: common set of interactions obtained by applying different LOI thresholds and a fixed PCC.fdr(0.1);

**E**: common set of interactions obtained by applying different LOI thresholds and a fixed PCC.fdr(0.05);

**F**: common set of interactions obtained by applying different LOI thresholds and a fixed PCC.fdr(0.005).

From Tables 1 and 2, it can be seen that the total number of previously published interactions remains almost entirely the same at different PCC.fdr(*q *) when the LOI.fdr(*q *) stringency is fixed at any level. Increasing LOI.fdr(*q *) has the effect of reducing the total number of predicted interactions without reducing the total number of previously published interactions. In every case, fixing the loosely restrictive PCC.fdr(0.1), the application of increasingly stringent LOI.fdr(*q *) improves the generated interaction network with respect to the percent of identified edges that have been previously published. This can be explained as follows. The LOI-scores for most of the published interactions in both sets are associated with substantially lower P-values in comparison to those unpublished pairs. More stringent LOI.fdr(*q *) can eliminate those unpublished pairs with relatively higher P-values for LOI-scores without excluding published pairs. The remaining unpublished pairs are likely to have LOI-scores with statistically significance and therefore are likely novel interacting pairs.

At the first impression, it seems that the 102-gene set performed better than the 999-gene set. There are, however, proportionately far fewer published interactions in the 999-gene set (0.09% of total possible interactions) than in the 102-gene set (1.9% of total possible interactions). For the 102-gene set, interaction set determined the thresholds LOI.fdr(0.05) and PCC.fdr(0.05) has 7.6% published interactions, which is about a 4-fold increase over the 1.9% published interactions out of all possible interactions. For the 999-gene set, the interaction set determined by the thresholds LOI.fdr(0.05) and PCC.fdr(0.05) has 2.0% published interactions, which is about a 22-fold increase over the 0.09% published interactions out of all possible interactions. Therefore, the result for the 999-gene set is actually a considerable improvement over the result for the 102-gene set. The method does a better job of finding published interactions in a search space where published interactions are proportionately much sparser.

Similarly, the distributions of GO BP annotations in the 102-gene and 999-gene sets have a significant effect on the generated interaction networks. The 102-gene set was selected to include known cell cycle regulated genes and thus tend to be enriched for a more limited selection of largely cell cycle related GO BP annotations. 26.4% of all possible gene interaction pairs in the 102-gene set share a GO BP annotation. The 999-gene set, on the other hand, was selected solely on the observed experimental data. Of all possible interaction pairs in the 999-gene set, there are 8.3% interaction pairs that share a GO BP annotation. A breakdown of the numbers of shared GO BP annotations for the identified interactions in the 999-gene set is shown in Table 3. Though the 102-gene set appears to perform better, identifying 39.4% (a 1.5-fold increase) interaction pairs that share a GO BP annotation compared to the 999-gene set with 23.0% (an almost 2.7-fold increase) at LOI.fdr(0.05) and PCC.fdr (0.05). Again, the method is actually doing a better job on the larger 999-gene set for finding matching GO BP annotation interaction pairs from a less enriched background.

**Table 3:** Breakdown of the numbers of shared GO BP annotations in the 999-gene set

**Number**	**GO ID**	**GO-BP Annotation**
71	GO:0006996	Organelle organization and biogenesis
53	GO:0006259	DNA metabolic process
40	GO:0007049	Cell cycle
25	GO:0006464	Protein modification
13	GO:0006950	Response to stress
8	GO:0009653	Anatomical structure morphogenesis
7	GO:0006350	Transcription
2	GO:0007126	Meiosis
1	GO:0007010	Cytoskeleton organization and biogenesis
1	GO:0007124	Pseudohyphal growth
1	GO:0006091	Generation of precursor metabolites and energy
1	GO:0000910	Cytokinesis
1	GO:0007114	Cell budding
1	GO:0042254	Ribosome biogenesis and assembly

## Discussion

### Possible novel interactions in the identified subnetworks

In the network identified at LOI.fdr(0.05) and PCC.fdr(0.05) for the 999-gene set, 15 previously published gene interactions were identified. The majority of these are interactions of transcriptional regulation, specifically the correct identification of transcription factors SWI4, FKH2, and FHL1 and several of their regulatory targets [22]. Additionally however, regulatory interactions other than simple transcription factors were found, such as kinase regulatory interaction mechanisms like GIN4 and KCC4 indirectly regulating expression SWE1 [32]. The correct identification of these known interactions adds credence to the possibility that some identified interactions which are not previously reported may be novel discoveries. These potential discoveries should be considered testable hypotheses for future experimentation.

There are a number of such potentially novel discoveries in the results from the interaction network of the 999-gene set. Some potential transcription factor regulatory interactions, specifically (1) FHL1 regulating DED1 and PFK1, (2) STB1 regulating CDC9, and (3) SWI4 regulating CDC9 all have regulation targets near appropriate transcription factor binding sites, though these interactions have not previously been characterized [33].

ASH1 can function as a part of a histone deactylase complex (HDAC) and is annotated as being likely to regulate many cell cycle related genes, though those interaction partners are not yet known [34]. Results from this analysis suggest that HSP150, PIL1, PIR1, PIR3, YLR049C, and YNL046W might be, directly or via intermediate gene interactions, regulated by ASH1. Though it is likely that many or most of these proposed novel interactions are false positives, all of them are immediately amenable to direct experimentation.

**Figure 1:**
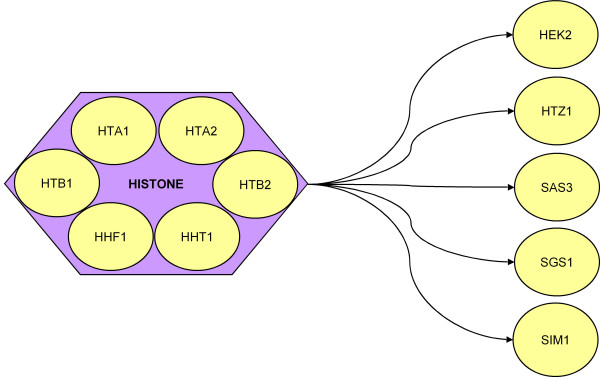
In this figure, interactions identified by analysis but not previously reported are observed to have potential biological interest. Genes and gene products are ovals, solid arrows are identified, directed interactions, dashed lines with arrows are more general interactions implied by this subnetwork. Purple box identifies those proteins that physically interact to form a functional unit. In this figure, cell cycle-regulated transcription factors (yellow) and protein kinase KCC4 concurrently regulates HSL1 and MNN1. MNN1 subsequently regulates PMT1-5. KCC4 and HSL1 (orange) are genes that associate with morphogenesis, septin checkpoints, and bud neck [32, 38-40]. MNN1 and PMT1-5 [green] are mannosyltransferases [41, 42]. These seemingly unrelated groups of genes and functions are united by the observation of Gladfelter, *et al. * [43] that the cell wall at the base of the bud is derived from mannose-rich glycoproteins that are delivered through the secretory pathway, and they suggest that septins target secretory vesicles to the base of the bud. Thus, the sub network identified here uniting cell cycle-regulating transcription factors with bud neck and septin checkpoint and mannosyltransferases, though not explicitly previously identified, is consistent with previous biological observations.

Figure 1 shows a subnetwork of interactions previously unidentified but of potential biological interest. All histone genes HHF1, HHF2, HHT1, HHT2, HTA1, HTA2, HTB1, and HTB2 are found to 'regulate' genes HEK2, HTZ1, SAS3, SGS1, and SIM1. The proteins encoded by these histone genes are known to form large complexes that bind genomic DNA into chromatin. Though it is unlikely that the histones directly regulate other genes, it may be reasonable to speculate that the histone complex are working together as a functional unit to interact with genes that control genome stability, chromosome packing, and cell-cycle specific DNA replication, making genes physically available to subsequent transcription.

**Figure 2:**
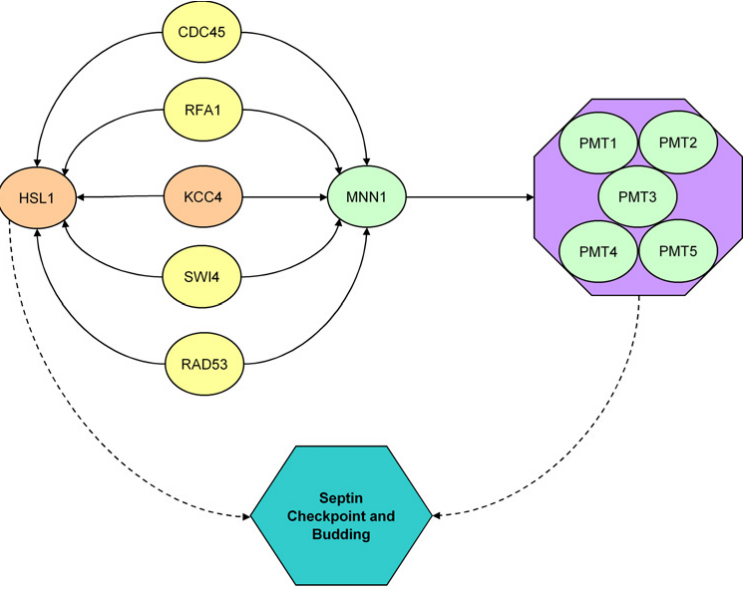
In this figure, interactions identified by analysis but not previously reported are observed to have potential biological interest. Genes and their coded proteins are ovals, arrows are identified, directed interactions, and the purple box includes those proteins that comprise histone complexes. All histone genes are identified to concurrently regulate other genes, correctly identifying HTA2, HTA1, HTB1, HTB2, HHT1, and HHF1 as a single functional unit. All regulated genes are associated with DNA-interacting proteins, which are logical functional partners to histone complexes. Regulated genes are: HEK2, associated with the regulation of telomeres [44, 45]; HTZ1, concerned with transcriptional regulation through heterochromatin structure [46]; SAS3, a cell cycle related histone acetyltransferase that is involved in transcriptional regulation [47]; SGS1, involved in maintenance of genome integrity and regulates chromosome synapsis and meiotic crossing over [48, 49]; SIM1, a cell cycle-regulated DNA replication gene [50]; ALK1, a cell cyle-regulated protein kinase involved with the response to DNA damage [51]; FPR4, a nuclear protein with GO annotations that include chromatin and histone associations [52-54]; and YPL141C, an unknown protein. The presence of YPL141C here however, suggests that its function may be related to chromatin structure, or cell cycle regulation.

In Figure 2, another set of unreported, but biologically interesting interactions identified by this method is presented. In this subnetwork, cell cycle related regulatory elements are determined to regulate the expression of two sets of genes: one is annotated to be associated with bud neck and septin checkpoint, and the other is annotated as mannosyltransferases. These two sets of genes and functions are united by the fact that the cell wall at the bud neck of the dividing yeast cell is derived from mannose-rich glycoproteins. Thus, this method correctly identifies a causal relationship between the regulation of genes that direct the yeast bud neck and the mannosyltransferase complexes that are needed to modify the proteins present at the bud neck.

### Novelty of the proposed method

The proposed method is robust. We tested the predictive ability of LOI-scores by removing 171 published interactions in the 102-gene set from the initial set of published 4,129 interacting pairs that was used for the calculation of LOI-scores for GO annotations. A similar predictive ability of LOI-scores for the 102-gene set was confirmed (results not shown). Our method has a number of advantages over other methods for determining genetic networks. Through the LOI-scores, gene interaction pairs that have not been previously identified can be uncovered bounded by biological expectation. A gene interaction pair need not have been previously observed, but only "be similar" in their GO MF annotation to previously observed interaction pairs.

Although a *q * level of 0.05 was used to identify interactions here, from Tables 1 and 2 it can be seen that at higher stringencies, superior networks with regard to percent of previously published and percent of interactions sharing a GO-BP annotation can be found. By adjusting the stringency of *q *, this method can generate smaller networks that are more likely to contain biologically significant interactions. Also the method can be adjusted to give greater weight to the LOI-scores or the PCCs, depending on a researcher's interest or nature of the data for analysis. Given a dataset of high quality or in a well-characterized system, the PCCs might prove most informative. In a largely unknown system or when the quality of the experimental data is poor, the LOI-score might be given greater weight. It was demonstrated that the addition of biological information improves the results. This makes this tool appropriate for hypothesis driven analysis of microarray data as the researcher can refine queries of the network by shaping the possibilities with biologically relevant constraints.

The method proposed here is also computationally inexpensive and could easily be scaled up to accommodate much larger datasets, while more mathematically intensive procedures require large amounts of processing time. Alternatively, the method here might be used as an efficient pre-screening of data, limiting the dataset to a smaller set with highly expected biological relevance before the data is given over to the analysis by more mathematically advanced methodologies, e.g. the Bayesian network approach.

### Choice of GO MF annotations

There are over two hundred thousand specific annotations in the GO ontology to choose from. However, the number of yeast genes at a particular annotation is an important factor in the appropriateness of the specific annotations used in this study. There must be a sufficient number of genes of a given annotation for useful statistical interpretation. If annotations are too general, resulting LOI scores would lack the ability to discriminate against large numbers of possible interaction pairs. Annotations that are too specific will apply only to a handful of genes and not have the ability to identify potentially novel interactions. The 23 SGD GO Slim MF annotations [30], not including the 'molecular function unknown', range in GO annotation level from second to ninth and contain between about less than 1% to about 12% of the yeast genome used here. Lower level GO MF terms do not necessarily include larger proportions of the genome. For example, it is observed for 'translation regulator activity' which is at level two and includes 1% of yeast genes, and 'lyase activity' which is at level three and includes 1.5% of genes (Table S1) [See additional file 1].

Therefore, these 23 annotations, which account a small percent of the entire GO annotations, are useful for the summarization of GO annotations at a genomic level when a broad classification of gene product function is required. Though the 23 annotations selected by the curators of the SGD may not be optimized for use in LOI-scores, these pre-selected annotations are an excellent place to begin with for the demonstration of the proposed method.

### The use of modified PCC

In this paper, the Pearson correlation coefficient was computed after applying the alignment based on a 1-time point shift of the gene expression of profile of a target to that of the corresponding regulator. Although this treatment is a simplification of the potential range of possible regulatory mechanisms, it was used to demonstrate that the LOI-method can improve on methods that use only expression data. This may result in underestimation of the performance of our proposed method when considering the common set of predicted interactions from both LOI-scores and PPC. More sophisticated alignment methods for the alignment of temporal profiles [35,36] may provide a better set of predicted interactions.

Our framework for computing LOI-scores is general. LOI-scores need not to be limited to either previously published interactions for an initial starting condition or GO MF for annotation of gene products. Any current large database of gene interactions could be used as the basis for LOI-score calculations and any appropriate gene product annotations could be used. The possibilities are limited only by the availability of data and ways that the most appropriate datasets and annotations can be applied to specific biological problems. It is useful to note that the single-celled yeast has a genome that contains around 6,000 genes. More complex, multicellular organisms like humans with around 30,000 genes will posses far more possible interactions and it is likely that finer grained annotations than those used for yeast will be necessary.

## Conclusion

We have proposed a method for generating new hypotheses from microarray data from a single microarray experiment using reported literature in PubMed. The likelihood of interaction for each pair of GO annotations of Molecular Function selected by the Saccharomyces Genome Database (SGD) GO Slim Mapper has been derived from the statistical analysis involved in the reported gene interaction pairs in literature. Combined with the analysis of correlation of microarray expression profiles, it has been demonstrated that the method can uncover existing and novel gene interactions in the analysis of the Saccharomyces cerevisiae cell cycle microarray data. The LOI-scores can be also used as a screen for possible gene interacting pairs. The output from this simple screening can be used as the input for other computationally intensive procedures, e.g. Bayesian network for inferring the gene networks.

## Methods

### PathwayAssist literature searching tool

There are many repositories of biological data available to researchers. For this study, the body of published, peer-reviewed literature in PubMed has been selected. This body of evidence, often derived from detailed studies of a handful of biological interactions is likely of higher quality than massive, false-positive prone screenings. PathwayAssist 3.0 analysis software by Ariadne Genomics [21] has been selected to take advantage of the data available in the published literature. PathwayAssist is a bioinformatics tool that identifies possible interactions between gene products through a natural language search algorithm of all available PubMed published abstracts. Given an input set of query genes or gene products, PathwayAssist searches the database of published abstracts, seeking instances in which genes are identified as interacting according to the information found in available PubMed abstracts. The nature of interactions ('expression', 'regulation', 'genetic interaction', 'binding', 'protein modification', and 'chemical modification' as defined in that software package) can be used to screen for specific types of interactions. The software returns the set of interactions with the PubMed references from which those interactions were identified. In this study, interaction types 'expression', 'regulation' and 'protein modification' were used for the extraction of the published interactions. By the definitions from PathwayAssist, 'Expression' is most likely to include interactions that affect gene expression levels, though it is populated largely by transcription factor regulators. 'Regulation' contains a mix of regulation of enzyme activity and regulation of gene expression. 'Protein modification' contains many specific examples of gene regulation through the activation or modification of regulatory proteins. No single interaction type specified by Pathway Assist can be expected to represent all relevant interactions that result in a measurable change in relative expression levels.

### Gene Ontology annotation

Not only is it necessary to acquire a large database of previously observed gene interactions, it is also necessary to be able to associate some biological knowledge on the gene products themselves. To impose biological knowledge on the set of gene products analyzed, annotation descriptions from the Gene Ontology (GO) were used. The GO annotation, maintained by the Gene Ontology Consortium [37] under the Open Biomedical Ontologies (OBO), is a structured, controlled vocabulary for describing gene products with regard to their biological process, molecular function, and cellular component.

GO annotation is an appealing resource for identifying biological information associated with a given gene product for use in automated analysis because its rigorously controlled vocabulary makes it applicable to computer-based searches. In this study, GO Molecular Function (MF) annotation is primarily used to impose biological knowledge on genes in the study set. MF annotation, a description of a gene product's biochemical activities, is the least ambiguous annotation and likely the most useful descriptor for how a gene product can physically interact with other gene products. MF annotation can also be estimated from sequence data, making it the most appropriate definition to describe gene products not well characterized by experimental data. A relatively non-specific MF annotation was selected for use in this analysis. In contrary, Biological Process (BP) annotation is more subjective and might actually inhibit the ability to identify novel interactions. An annotation of a given BP should not preclude the possibility that the gene product can act in other, as yet uncharacterized biological processes. Cellular Component GO annotations, though possibly can be well estimated from sequence data, is not likely to describe gene products in a way that is appropriate to their interactions with other gene products. Therefore, BP annotation was used for validation of biological plausibility of the identified interactions in our study.

The GO Slim Mapper [30] in Saccharomyces Genome Database (SGD) was used to extract 23 GO MF annotations with broad descriptions of molecular functions. This set of specific annotations was selected by the curators of the SGD at the Department of Genetics of the School of Medicine, Stanford University. The 23 GO MF annotations are listed in Table 4.

**Table 4:** The 23 GO MF annotations used in the proposed method

**GO ID**	**GO MF Annotation**
GO:0003674	Molecular function unknown
GO:0016740	Transferase activity
GO:0016787	Hydrolase activity
GO:0030528	Transcription regulator activity
GO:0005198	Structural molecule activity
GO:0005215	Transporter activity
GO:0005515	Protein binding
GO:0003677	DNA binding
GO:0003723	RNA binding
GO:0016491	Oxidoreductase activity
GO:0030234	Enzyme regulator activity
GO:0004672	Protein kinase activity
GO:0006874	Ligase activity
GO:0008233	Peptidase activity
GO:0016779	Nucleotidyltransferase activity
GO:0004871	Signal transducer activity
GO:0004386	Helicase activity
GO:0006874	Ligase activity
GO:0045182	Translation regulator activity
GO:0016853	Isomerase activity
GO:0004721	Phosphoprotein phosphatase activity
GO:0003774	Moter activity
Other	Other

Molecular function unknown (GO: 0003674) from the GO slim annotation terms was included in the set of annotations for the calculation of LOI scores. In the yeast genome, there are nearly two thousand genes (about one third of the genome) that have the annotation 'molecular function' according to the SGD. From the set of interactions identified by 'Pathway Studio', nearly 15% involve a target or regulator of the annotation 'molecular function unknown'. Although a gene product's annotation may not yet characterized, it still can participate in biologically meaningful interactions for the analysis of microarray data. The consideration of the frequency at which a gene with undermined function is involved in an interacting partner with known annotations allows the LOI-method for the potential identification of useful biological interactions even when the function of one of the genes remains unknown. Ideally, as biological information of an organism's entire set of expressed proteins becomes known, the necessity of including an annotation of unknown molecular function will vanish.

### Identify "true" interactions

Yeast cell cycle microarray data has the advantage of being a well-studied biological phenomenon and is also one of the key datasets from which many studies of gene interaction network draw.

In order to test the accuracy of the method proposed here, it is necessary to have some expectations as to the true gene interaction network. However, the 'true' gene network is not known and depends upon the chosen definition of gene interaction. For this study, the 'true' interactions were derived from the database of PathwayAssist by submitting the list of genes and querying for instances of published interactions between these genes limited to interaction types 'expression' and 'regulation'. It should be noted that this is not a so-called 'golden standard' set for a true evaluation of the learning outcome. Nevertheless, this list of previously published gene interaction pairs can be reasonably considered to be 'true' gene interactions in this dataset. One hundred seventy one and 729 previously published gene interactions were identified respectively in the 102-gene set and in the set of 999 cyclically expressed genes.

### Generate LOI-scores for gene annotations

Two thousand four hundred and fifty seven yeast genes were selected from the Saccharomyces cerevisiae database of PathwayAssist 3.0 and used to identify 4,192 directed gene interaction pairs of interaction types "Expression", "Regulation", and "Protein Modification" as defined in that software package. These gene interactions are suggested by 4,446 observed facts from the automated literature search. The same set of 2,457 yeast genes was annotated with one of the 23 GO MF annotations by the SGD GO Slim Mapper [30].

**Figure 3:**
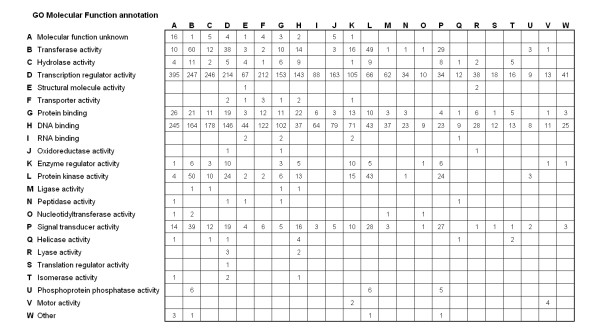
**Numbers of pairs of GO MF annotations counted in the reported interactions**. 2,457 yeast genes used to identify 4,192 directed gene interaction pairs by PathwayAssist. The set of 2,457 yeast genes was annotated with one of 23 GO molecular function annotation. This yielded 5,014 annotated interaction pairs.

Using the 4,192 interacting gene pairs, the GO MF annotations of the regulator and the target genes were considered. Five thousand and fourteen pairs of a regulator of one annotation modulating the expression of an annotated target were observed. Due to the fact that some gene products have multiple GO annotations, this exceeds the 4,192 interaction pairs in the dataset. The number of times a specific GO annotation is observed to modulate the expression of another specific GO annotation was counted (Figure 3). For example, of the 5,014 pairs of interactions, 163 instances were observed for regulators of the annotation 'transcription regulator activity' regulating genes of the annotation 'oxidoreductase activity'.

**Figure 4:**
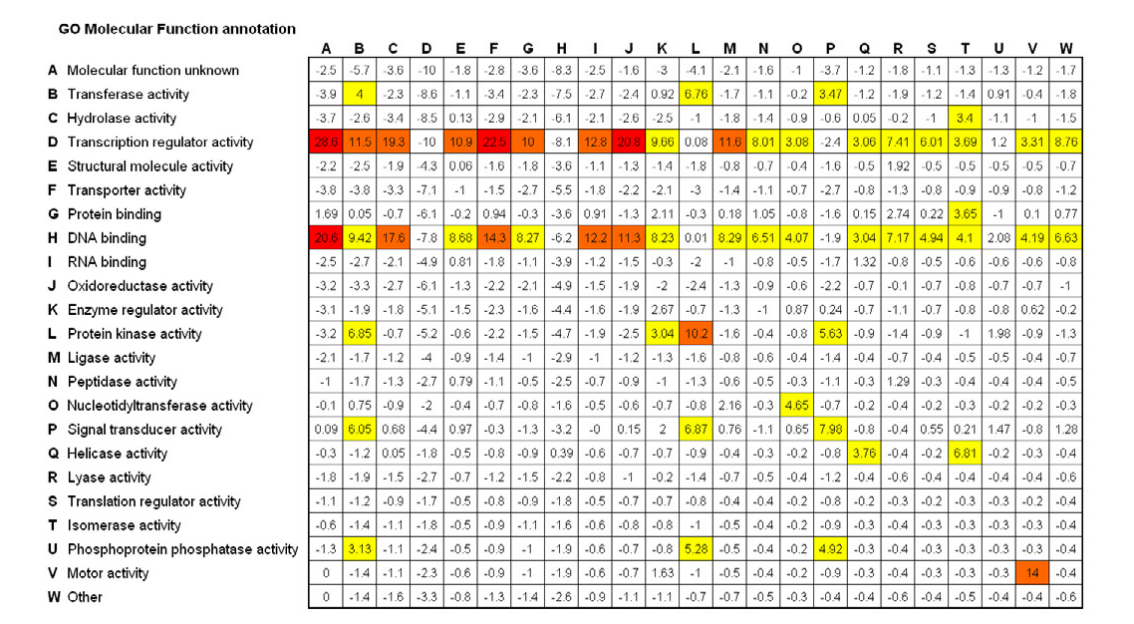
Likelihood of interaction for a pair of GO MF annotations.

The distribution of GO annotations in the table of gene interaction pair is heterogeneous, dependent on both the number of observations of gene interactions and the frequency of GO annotations in the gene interactions. From this table of observations, it is necessary to determine if pairs of GO annotations found in gene interactions at a frequency is greater than random, given the distribution of those GO annotations in the observed data. To address this question, the following procedure was used to calculate the distribution of LOI-scores for pairs of GO annotations for randomly generated interaction pairs. At each iteration, 4,192 gene pairs were drawn from the set of published gene interactions with a randomly selected gene for the regulator and randomly selected gene for the target. For each permutation, the calculated number of times a specific GO annotation regulated the expression of another specific GO annotation was recorded. An average and standard deviation for randomly observed GO pairs were generated from all 10,000 iterations. An LOI-score for each GO annotation pair was generated as a Z-score:

LOI_*ij *_= (O_*ij *_- X_*ij *_)/S_*ij *_,

where LOI_ij _is the Z-score for interaction GO molecular function annotation pair GO_i _and GO_j_, O_ij _is the number of times that genes of annotations GO_i _and GO_j _were observed in regulator to target relationships in the literature-derived dataset. X_ij _and S_ij _are the average number of times and standard deviation respectively from the procedure for interaction pair of annotations GO_i _and GO_j_. The LOI-scores calculated from the above procedure are shown in Figure 4. A negative LOI_ij_-score indicates that a particular GO-annotation pair occurs less frequently than expected by random chance. A positive LOI-score indicates an interaction between GO annotations occurs more frequently than expected at random. A score near zero indicates that the frequency occurs at a level near that expected by random. As a general estimation of the utility of the information encoded in this figure, it is observed that the GO annotations for a pair of 'transcription regulator activity' and 'DNA binding activity' have the highest LOI-scores, as would be reasonably expected for genes annotated as regulators of expression.

### Generate LOI-scores for gene interactions

The table of calculated LOI-scores for GO annotation pairs was used to generate a matrix of LOI-scores for all possible gene interaction pairs in the subsets of the yeast cell cycle microarray data. The 23 annotations described previously were applied to the genes in the subsets. For a possible interaction pair between two genes, their annotations were used for the assignment of a LOI-score for the likelihood of that interaction from the previously calculated table of LOI-scores. If a gene possessed multiple annotations, then a LOI-score was averaged between all possible pairs of annotations for a given potential interaction pair.

### Calculate cyclic correlation coefficients

The cyclic correlation coefficient (CCC) is defined as follows.

CCC = MAX { PEARSON ((*X *_*1 *_,...,*X *_*t *_), (*X *_*t *+*1 *_,...,*X *_*2t *_)) }; *t * = 9, 8, 7, 6,

where PEARSON is the Pearson correlation coefficient between two sets of values, *X *_*i *_is a gene's expression at time *i *, *t * is the size of window for comparing one length of gene expression values with another. Windows of size 9, 8, 7, and 6 were used for this analysis. Windows of these sizes should be suitable for finding cyclic patterns in 18 time points with more than two, but less than three complete cycles measured.

### The modified Pearson correlation coefficient (PCC)

The modified Pearson correlation coefficient is defined based on one time shift. For each pair of regulator profile X and target profile Y, the coefficient is defined as

PCC = PEARSON((*X *_*1 *_,*X *_*2 *_,...,*X *_*n *-*1 *_), (*Y *_*2 *_,*Y *_*3 *_,...,*Y *_*n *_)),

where *n * is the number of time points.

### Assign P-values to LOI-scores and PCCs

In order to determine the statistically significant gene interaction pairs, P-values need to be assigned to LOI-scores of gene interaction pairs and PCCs. The P-values for LOI-scores were assigned based on the assumptions that the LOI-score for a pair of interaction is normally distributed (Figure S1- Figure S3) [see additional file 2].

To obtain *P *-values for PCC*s *, the following steps were performed.

1) PCCs were obtained for the observed gene expression profiles.

2) For *b * = 1,...,*B *, where *B * is a prescribed number.

a. Generate a random data set by permuting the gene expression values over time points.

b. Calculate PCCijb
 MathType@MTEF@5@5@+=feaafiart1ev1aaatCvAUfKttLearuWrP9MDH5MBPbIqV92AaeXatLxBI9gBaebbnrfifHhDYfgasaacH8akY=wiFfYdH8Gipec8Eeeu0xXdbba9frFj0=OqFfea0dXdd9vqai=hGuQ8kuc9pgc9s8qqaq=dirpe0xb9q8qiLsFr0=vr0=vr0dc8meaabaqaciaacaGaaeqabaqabeGadaaakeaacqqGqbaucqqGdbWqcqqGdbWqdaqhaaWcbaGaemyAaKMaemOAaOgabaGaemOyaigaaaaa@341F@ for each pair of gene expression profiles in the random set.

3) Calculate the empirical *P *-value for each PCC_*ij *_:

P−value=∑b=1BI(PCCijb>PCCij)B,
 MathType@MTEF@5@5@+=feaafiart1ev1aaatCvAUfKttLearuWrP9MDH5MBPbIqV92AaeXatLxBI9gBaebbnrfifHhDYfgasaacH8akY=wiFfYdH8Gipec8Eeeu0xXdbba9frFj0=OqFfea0dXdd9vqai=hGuQ8kuc9pgc9s8qqaq=dirpe0xb9q8qiLsFr0=vr0=vr0dc8meaabaqaciaacaGaaeqabaqabeGadaaakeaacqWGqbaucqGHsislcqWG2bGDcqWGHbqycqWGSbaBcqWG1bqDcqWGLbqzcqGH9aqpdaWcaaqaamaaqahabaGaemysaKKaeiikaGccbaGae8huaaLae83qamKae83qam0aa0baaSqaaiabdMgaPjabdQgaQbqaaiabdkgaIbaakiabg6da+iab=bfaqjab=neadjab=neadnaaBaaaleaacqWGPbqAcqWGQbGAaeqaaOGaeiykaKcaleaacqWGIbGycqGH9aqpcqaIXaqmaeaacqWGcbGqa0GaeyyeIuoaaOqaaiabdkeacbaacqGGSaalaaa@50C6@

where *I *(·) is an indicator function which is defined as 1 if the condition inside the parenthesis is true, 0 otherwise.

### Multiple hypothesis testing

The procedure of Benjamini and Hochberg [31] was used for multiple hypothesis testing for both LOI-scores and PCCs. The steps are summarized as follows:

1. Specify a false discovery rate (fdr) *q *.

2. Sort the list of *P *-values in increasing order.

3. Find index y_0 _where P-values less than index y_0 _is considered significant:

y0=max⁡{y:py≤yqm,y=1,...,m}
 MathType@MTEF@5@5@+=feaafiart1ev1aaatCvAUfKttLearuWrP9MDH5MBPbIqV92AaeXatLxBI9gBaebbnrfifHhDYfgasaacH8akY=wiFfYdH8Gipec8Eeeu0xXdbba9frFj0=OqFfea0dXdd9vqai=hGuQ8kuc9pgc9s8qqaq=dirpe0xb9q8qiLsFr0=vr0=vr0dc8meaabaqaciaacaGaaeqabaqabeGadaaakeaacqWG5bqEdaWgaaWcbaGaeGimaadabeaakiabg2da9iGbc2gaTjabcggaHjabcIha4jabcUha7jabdMha5jabcQda6iabdchaWnaaBaaaleaacqWG5bqEaeqaaOGaeyizIm6aaSaaaeaacqWG5bqEcqWGXbqCaeaacqWGTbqBaaGaeiilaWIaemyEaKNaeyypa0JaeGymaeJaeiilaWIaeiOla4IaeiOla4IaeiOla4IaeiilaWIaemyBa0MaeiyFa0haaa@4D36@

where *m * is the total number of hypotheses.

## Authors' contributions

The main framework was formed by PL and YD. PL extracted data and performed biological analysis of the outcome. EA and GC participated in the computation. YD supervised overall project. All authors have read and approved the final manuscript.

## Supplementary Material

Additional file 1 Distribution of number of annotated genes. The data provides the distribution of numbers of annotated genes distribution of at the selected 23 GO MF annotations by GO Slim Mapper. Click here for file

Additional file 2 Examples of histograms of GO MF annotation pairs. The figures provide evidences that GO annotation pairs follow normal distribution. Click here for file

## References

[B1] Liang S, Fuhrman S, Somogyi R (1998). Reveal, a general reverse engineering algorithm for inference of genetic network architectures. Pac Symp Biocomput.

[B2] Murphy KP, Mian S (1999). Modeling gene expression data using dynamic Bayesian networks. Technical report.

[B3] Ideker TE, Thorsson V, Karp RM (2000). Discovery of regulatory interactions through perturbation: inference and experimental design. Pac Symp Biocomput.

[B4] D'Haeseleer P, Liang S, Somogyi R (2000). Genetic network inference: from co-expression clustering to reverse engineering. Bioinformatics.

[B5] Friedman N, Linial M, Nachman I, Pe'er D (2000). Using Bayesian networks to analyze expression data. J Comput Biol.

[B6] Pe'er D, Regev A, Elidan G, Friedman N (2001). Inferring subnetworks from perturbed expression profiles. Bioinformatics.

[B7] Hartemink AJ, Gifford DK, Jaakkola TS, Young RA (2001). Using graphical models and genomic expression data to statistically validate models of genetic regulatory networks. Pac Symp Biocomput.

[B8] Wagner A (2002). Estimating coarse gene network structure from large-scale gene perturbation data. Genome Res.

[B9] Yeung MK, Tegner J, Collins JJ (2002). Reverse engineering gene networks using singular value decomposition and robust regression. Proc Natl Acad Sci U S A.

[B10] Wang W, Cherry JM, Botstein D, Li H (2002). A systematic approach to reconstructing transcription networks in Saccharomycescerevisiae. PNAS.

[B11] Stuart JM, Segal E, Koller D, Kim SK (2003). A Gene-Coexpression Network for Global Discovery of Conserved Genetic Modules. Science.

[B12] Segal E, Shapira M, Regev A, Pe'er D, Botstein D, Koller D, Friedman N (2003). Module networks: identifying regulatory modules and their condition-specific regulators from gene expression data. Nat Genet.

[B13] Liao JC, Boscolo R, Yang YL, Tran LM, Sabatti C, Roychowdhury VP (2003). Network component analysis: Reconstruction of regulatory signals in biological systems. PNAS.

[B14] Hartemink AJ, Gifford DK, Jaakkola TS, Young RA (2002). Combining location and expression data for principled discovery of genetic regulatory network models. Pac Symp Biocomput.

[B15] Gao F, Foat BC, Bussemaker HJ (2004). Defining transcriptional networks through integrative modeling of mRNA expression and transcription factor binding data. BMC Bioinformatics.

[B16] Tanay A, Sharan R, Kupiec M, Shamir R (2004). Revealing modularity and organization in the yeast molecular network by integrated analysis of highly heterogeneous genomewide data. PNAS.

[B17] Rajagopalan D, Agarwal P (2005). Inferring pathways from gene lists using a literature-derived network of biological relationships. Bioinformatics.

[B18] Herrgard MJ, Covert MW, Palsson B (2003). Reconciling Gene Expression Data With Known Genome-Scale Regulatory Network Structures. Genome Res.

[B19] Chrisman L, Langley P, Bay S, Pohorille A (2003). Incorporating biological knowledge into evaluation of causal regulatory hypotheses.

[B20] Jenssen TK, Laegreid A, Komorowski J, Hovig E (2001). A literature network of human genes for high-throughput analysis of gene expression. Nat Genet.

[B21] Nikitin A, Egorov S, Daraselia N, Mazo I (2003). Pathway studio--the analysis and navigation of molecular networks. Bioinformatics.

[B22] Spellman PT, Sherlock G, Zhang MQ, Iyer VR, Anders K, Eisen MB, Brown PO, Botstein D, Futcher B (1998). Comprehensive identification of cell cycle-regulated genes of the yeast Saccharomyces cerevisiae by microarray hybridization. Molecular biology of the cell.

[B23] Zou M, Conzen SD (2005). A new dynamic Bayesian network (DBN) approach for identifying gene regulatory networks from time course microarray data. Bioinformatics.

[B24] Zhao LP, Prentice R, Breeden L (2001). Statistical modeling of large microarray data sets to identify stimulus-response profiles. Proceedings of the National Academy of Sciences of the United States of America.

[B25] Johansson D, Lindgren P, Berglund A (2003). A multivariate approach applied to microarray data for identification of genes with cell cycle-coupled transcription. Bioinformatics.

[B26] Lu X, Zhang W, Qin ZS, Kwast KE, Liu JS (2004). Statistical resynchronization and Bayesian detection of periodically expressed genes. Nucleic acids research.

[B27] Luan Y, Li H (2004). Model-based methods for identifying periodically expressed genes based on time course microarray gene expression data. Bioinformatics.

[B28] Filkov V, Skiena S, Zhi J (2002). Analysis techniques for microarray time-series data. J Comput Biol.

[B29] LOI-score Calculator. http://array.bioengr.uic.edu/LOI.zip.

[B30] GO Slim Mapper. http://db.yeastgenome.org/cgi-bin/GO/goTermMapper.

[B31] Benjamini Y, Yekutieli D (2001). The control of the false discovery rate in multiple testing under dependency. Annals of Sattistics.

[B32] Okuzaki D, Watanabe T, Tanaka S, Nojima H (2003). The Saccharomyces cerevisiae bud-neck proteins Kcc4 and Gin4 have distinct but partially-overlapping cellular functions. Genes Genet Syst.

[B33] Harbison CT, Gordon DB, Lee TI, Rinaldi NJ, Macisaac KD, Danford TW, Hannett NM, Tagne JB, Reynolds DB, Yoo J, Jennings EG, Zeitlinger J, Pokholok DK, Kellis M, Rolfe PA, Takusagawa KT, Lander ES, Gifford DK, Fraenkel E, Young RA (2004). Transcriptional regulatory code of a eukaryotic genome. Nature.

[B34] Carrozza MJ, Florens L, Swanson SK, Shia WJ, Anderson S, Yates J, Washburn MP, Workman JL (2005). Stable incorporation of sequence specific repressors Ash1 and Ume6 into the Rpd3L complex. Biochimica et Biophysica Acta (BBA) - Gene Structure and Expression.

[B35] He F, Zeng AP (2006). In search of functional association from time-series microarray data based on the change trend and level of gene expression. BMC Bioinformatics.

[B36] Ernst J, Bar-Joseph Z (2006). STEM: a tool for the analysis of short time series gene expression data. BMC Bioinformatics.

[B37] Gene Ontology Consortium (2001). Creating the gene ontology resource: design and implementation. Genome research.

[B38] Longtine MS, Theesfeld CL, McMillan JN, Weaver E, Pringle JR, Lew DJ (2000). Septin-dependent assembly of a cell cycle-regulatory module in Saccharomyces cerevisiae. Mol Cell Biol.

[B39] Okuzaki D, Nojima H (2001). Kcc4 associates with septin proteins of Saccharomyces cerevisiae. FEBS letters.

[B40] Shulewitz MJ, Inouye CJ, Thorner J (1999). Hsl7 localizes to a septin ring and serves as an adapter in a regulatory pathway that relieves tyrosine phosphorylation of Cdc28 protein kinase in Saccharomyces cerevisiae. Mol Cell Biol.

[B41] Girrbach V, Strahl S (2003). Members of the evolutionarily conserved PMT family of protein O-mannosyltransferases form distinct protein complexes among themselves. The Journal of biological chemistry.

[B42] Lussier M, Sdicu AM, Bussey H (1999). The KTR and MNN1 mannosyltransferase families of Saccharomyces cerevisiae. Biochimica et biophysica acta.

[B43] Gladfelter AS, Kozubowski L, Zyla TR, Lew DJ (2005). Interplay between septin organization, cell cycle and cell shape in yeast. Journal of cell science.

[B44] Irie K, Tadauchi T, Takizawa PA, Vale RD, Matsumoto K, Herskowitz I (2002). The Khd1 protein, which has three KH RNA-binding motifs, is required for proper localization of ASH1 mRNA in yeast. Embo J.

[B45] Denisenko O, Bomsztyk K (2002). Yeast hnRNP K-like genes are involved in regulation of the telomeric position effect and telomere length. Mol Cell Biol.

[B46] Meneghini MD, Wu M, Madhani HD (2003). Conserved histone variant H2A.Z protects euchromatin from the ectopic spread of silent heterochromatin. Cell.

[B47] Howe L, Auston D, Grant P, John S, Cook RG, Workman JL, Pillus L (2001). Histone H3 specific acetyltransferases are essential for cell cycle progression. Genes & development.

[B48] Rockmill B, Fung JC, Branda SS, Roeder GS (2003). The Sgs1 helicase regulates chromosome synapsis and meiotic crossing over. Curr Biol.

[B49] Versini G, Comet I, Wu M, Hoopes L, Schwob E, Pasero P (2003). The yeast Sgs1 helicase is differentially required for genomic and ribosomal DNA replication. Embo J.

[B50] Mouassite M, Guerin MG, Camougrand NM (2000). The SUN family of Saccharomyces cerevisiae: the double knock-out of UTH1 and SIM1 promotes defects in nucleus migration and increased drug sensitivity. FEMS microbiology letters.

[B51] Nespoli A, Vercillo R, di Nola L, Diani L, Giannattasio M, Plevani P, Muzi-Falconi M (2006). Alk1 and Alk2 are two new cell cycle-regulated haspin-like proteins in budding yeast. Cell cycle (Georgetown, Tex.

[B52] Dolinski K, Muir S, Cardenas M, Heitman J (1997). All cyclophilins and FK506 binding proteins are, individually and collectively, dispensable for viability in Saccharomyces cerevisiae. Proceedings of the National Academy of Sciences of the United States of America.

[B53] Kuzuhara T, Horikoshi M (2004). A nuclear FK506-binding protein is a histone chaperone regulating rDNA silencing. Nature structural & molecular biology.

[B54] Nelson CJ, Santos-Rosa H, Kouzarides T (2006). Proline isomerization of histone H3 regulates lysine methylation and gene expression. Cell.

