# Supplementary material for: A statistical method to incorporate biological knowledge for generating testable novel gene regulatory interactions from microarray experiments
Source: BMC Bioinformatics. 2007 Aug 29;8:317. doi: 10.1186/1471-2105-8-317 (PMC2082045; doi:10.1186/1471-2105-8-317)
Supplement: Additional file 1 — Distribution of number of annotated genes. The data provides the distribution of numbers of annotated genes distribution of at the selected 23 GO MF annotations by GO Slim Mapper. [file 1471-2105-8-317-S1.doc]

Table S1. Distribution of annotated genes at the selected 23 GO MF annotations by GO Slim Maper.

| **Level** | **GO ID** | **GO-Slim MF Annotations** | **% Genes Annotated** |
| --- | --- | --- | --- |
| **2** | GO:0003774 | Motor activity | 0.3% |
| **7** | GO:0004721 | Phosphoprotein phosphatase activity | 0.8% |
| **3** | GO:0016853 | Isomerase activity | 0.9% |
| **2** | GO:0045182 | Translation regulator activity | 1.0% |
| **3** | GO:0004871 | Signal transducer activity | 1.1% |
| **5** | GO:0016779 | Nucleotidyltransferase activity | 1.4% |
| **3** | GO:0016829 | Lyase activity | 1.5% |
| **3** | GO:0004386 | Helicase activity | 1.6% |
| **4** | GO:0008233 | Peptidase activity | 2.1% |
| **3** | GO:0006874 | Ligase activity | 2.2% |
| **6** | GO:0004672 | Protein kinase activity | 2.3% |
| **2** | GO:0030234 | Enzyme regulator activity | 3.1% |
| **4** | GO:0003677 | DNA binding | 3.7% |
| **4** | GO:0003723 | RNA binding | 4.0% |
| **3** | GO:0016491 | Oxidoreductase activity | 4.5% |
| **2** | GO:0030528 | Transcription regulator activity | 5.7% |
| **2** | GO:0005198 | Structural molecule activity | 5.9% |
| **2** | GO:0005215 | Transporter activity | 7.1% |
| **3** | GO:0005515 | Protein binding | 7.2% |
| **3** | GO:0016740 | Transferase activity | 11.6% |
| **3** | GO:0016787 | Hydrolase activity | 12.5% |
| -- | other | Other | 2.4% |
| **1** | GO:0003674 | Oolecular function unknown | 34.8% |

Level : this is the level of the annotation on the ontology tree of molecular function.
