# Supplementary material for: A statistical method to incorporate biological knowledge for generating testable novel gene regulatory interactions from microarray experiments
Source: BMC Bioinformatics. 2007 Aug 29;8:317. doi: 10.1186/1471-2105-8-317 (PMC2082045; doi:10.1186/1471-2105-8-317)

Examples of histograms of GO MF annotation pairs

(1) Hydrolase activity and Transcription regulator activity (Figure S1)

(2) Transcription regulator activity and DNA binding (Figure S2)

(3) Transcription regulator activity and Transporter activity (Figure S3)

are shown below. The values of R^2^ are correlation coefficients when they are fitted with normal distribution.

Histograms of all the other GO MF annotation pairs are omitted.

Figure S1. Histogram of the observation after 10000 times random samplings for the GO pair Hydrolase activity and Transcription regulator activity (R^2^=.986)


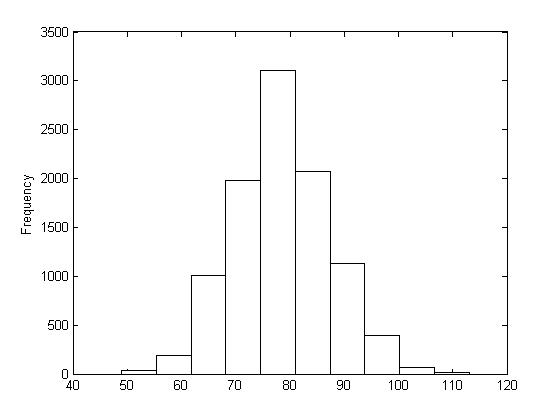


Figure S2. Histogram of the observation after 10000 times random samplings for the GO pair Transcription regulator activity and DNA binding (R^2^=.998)


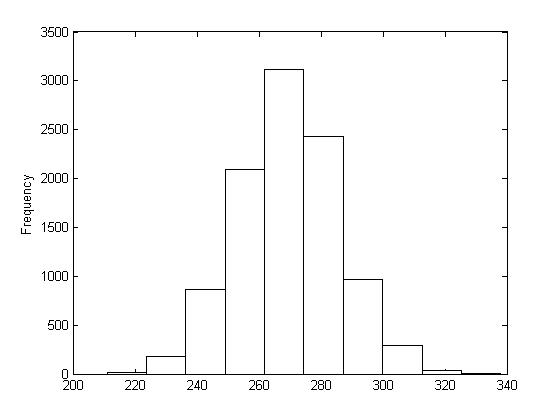


Figure S3. Histogram of the observation after 10000 times random samplings for GO pair Transcription regulator activity and Transporter activity (R^2^=.993)


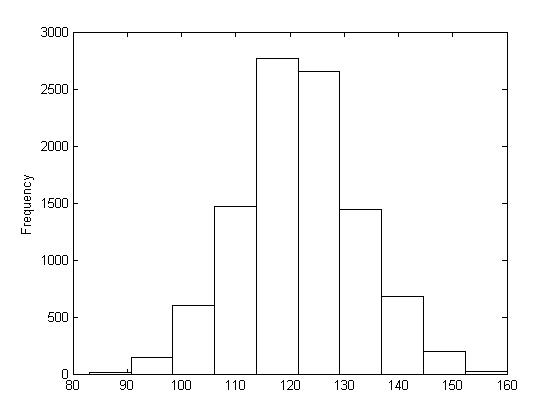

Supplement: Additional file 2 — Examples of histograms of GO MF annotation pairs. The figures provide evidences that GO annotation pairs follow normal distribution. [file 1471-2105-8-317-S2.doc]
